# Design of *Trypanosoma rangeli* sialidase mutants with improved trans-sialidase activity

**DOI:** 10.1371/journal.pone.0171585

**Published:** 2017-02-03

**Authors:** Christian Nyffenegger, Rune Thorbjørn Nordvang, Carsten Jers, Anne S. Meyer, Jørn Dalgaard Mikkelsen

**Affiliations:** Center for BioProcess Engineering, Department of Chemical and Biochemical Engineering, Technical University of Denmark, Kgs. Lyngby, Denmark; University of Toulouse—Laboratoire d'Ingénierie des Systèmes Biologiques et des Procédés, FRANCE

## Abstract

A sialidase (EC 3.2.1.18) from the non-pathogenic *Trypanosoma rangeli*, TrSA, has been shown to exert trans-sialidase activity after mutation of five specific amino acids in the active site (M96V, A98P, S120Y, G249Y, Q284P) to form the so-called TrSA_5mut_ enzyme. By computational and hypothesis driven approaches additional mutations enhancing the trans-sialidase activity have been suggested. In the present work, we made a systematic combination of these mutations leading to seven new variants of the *T*. *rangeli* sialidase, having 6–16 targeted amino acid mutations. The resulting enzyme variants were analyzed via kinetics for their ability to carry out trans-sialidase reaction using CGMP and D-lactose as substrates. The sialidase variants with 15 and 16 mutations, respectively, exhibited significantly improved trans-sialidase activity for D-lactose sialylation. Our results corroborate, that computational studies of trans-glycosylation can be a valuable input in the design of novel trans-glycosidases, but also highlight the importance of experimental validation in order to assess the performance. In conclusion, two of the seven mutants displayed a dramatic switch in specificity from hydrolysis towards trans-sialylation and constitute the most potent trans-sialidase mutants of TrSA described in literature to date.

## Introduction

Human milk oligosaccharides (HMO) are comprised of a backbone of D-lactose elongated with N-acetyl-D-glucosamine and D-galactose which can be decorated with L-fucose and/or sialic acid (SA) residues. They are present in human milk in concentrations of 12–14 g/L and have been attributed a number of beneficial effects including acting as prebiotics, preventing pathogen adhesion, modulation of immune and cell responses, and supplying nutrients for brain development [1]. Amongst the subset of sialylated HMOs, 3’-sialyl-D-lactose (3’SL), has been shown to reduce adhesion and invasion of *Escherichia coli in vitro* [1–3]. Moreover, 6’-sialyl-D-lactose was recently shown to reduce food allergy symptoms in mice [4].

While HMOs are abundant in human milk, only trace amounts are present in infant formula based on bovine milk. Therefore the production of HMOs is receiving increased attention, both for commercial use as well as for functional studies of individual HMOs. The trans-sialidase from the human pathogen *Trypanosoma cruzi* (TcTS) exhibits high trans-sialidase activity and low hydrolytic activity and has previously been used for enzymatic glycan sialylation [5]. However, for industrial production of food-grade HMOs, it is a drawback that the enzyme constitutes an important virulence factor in *T*. *cruzi* [6]. Redesigning mutants of the non-pathogenic *T*. *rangeli* sialidase (TrSA), which possesses relatively low trans-sialidase activity, provides an attractive alternative for application in bioconversion processes. TrSA has 70% sequence identity to TcTS, has the same overall tertiary structure, and both enzymes have been extensively characterized by biochemical, mutational and structural studies. TrSA and TcTS are both members of the glycoside hydrolase family 33 and share a common double displacement mechanism with a tyrosine as catalytic nucleophile [7,8]. Despite the high sequence identity between TcTS and TrSA, it has proven difficult to pinpoint the amino acids necessary for trans-sialidase activity [9–11]. Paris and co-workers showed that introduction of the five mutations M96V, A98P, S120Y, G249Y, and Q284P in TrSA (TrSA_5mut_) is needed to confer detectable trans-sialidase activity (0.9% compared to TcTS) and either of the mutations I37L or G342A leads to a 12-fold improvement in trans-sialidase activity [10]. TrSA_5mut_ I37L (TrSA_6mut_) has been expressed in *Pichia pastoris* and was used for synthesis of 3’SL as well as other sialylated glycans [12]. In a subsequent study, we identified a highly charged loop region (amino acids 197–203) 14 Å from the acceptor binding site in TcTS [11]. Introducing the seven-amino acid motif in TrSA_6mut_ (to generate TrSA_13mut_) reduces the hydrolytic activity without significantly affecting the trans-sialidase activity, thus allowing higher product yields due to reduced sialyl-donor and product de-sialylation [11].

In a recent study, free-energy profiles for the conversion of the Michaelis complex (MC) into the covalent intermediate (CI) were computed for TcTS, TrSA and TrSA_5mut_ demonstrating that the covalent intermediate (CI) in TcTS is less stable than in TrSA [13]. The consequence of higher stability of the CI is a higher barrier for the reverse reaction, *i*.*e*. the step necessary for trans-sialidase activity [13]. Using energy decomposition analysis, the contribution to CI stability of individual active site residues in TrSA_5mut_ was calculated. The five most CI stabilizing residues include three of the five mutated residues (D97, W313, and E358) indicating slightly different positioning as well as two residues not identical with the respective residues in TcTS (F59 and D285, which correspond to N58 and G284 in TcTS). T39A was predicted to change the stability of the CI indirectly by modifying the conformation of E358 [13]. The mutations I37L and G342A, also identified by Paris and co-workers [10], were proposed to increase the participation of the nucleophile at the transition state by improving the mobility of the nucleophile [13]. Collectively, they proposed that introducing the mutations I37L, T39A, F59N, D285G and G342A in TrSA_5mut_ would yield an enzyme with high trans-sialidase activity. This, however, was not confirmed experimentally.

Development of trans-glycosidases from natural hydrolases aims at increasing the ratio of trans-glycosidase vs hydrolase activity. In general, both donor and product can be subject to the hydrolytic activity, which leads to reduced product yields as well as contamination with monosaccharides (in case of exo-glycosidases). It is thus paramount to eliminate or reduce the hydrolytic activity. Reaction condition design, and especially the use of high substrate concentrations, has been shown to favour trans-glycosylation activity over hydrolase activity for trans-sialidases and other trans-glycosidases in e.g. galactooligosaccharide production [12,14]. Insights from trans-glycosidase engineering indicate that mutations in the donor sugar binding site compared to mutation in the acceptor binding site in general are more likely to decrease hydrolytic activity [15].

In this paper, the findings of the three studies conducted by Paris *et al*., Jers *et al*., and Pierdominici-Sottile *et al*. [10,11,13] were integrated in an effort to design a sialidase derived from *T*. *rangeli* with improved trans-sialidase activity and/or reduced hydrolytic activity. Among the mutations analyzed in this study, the structural mutation I37L, the energetic mutations T39A, and D286G are located in the donor binding site, whereas the energetic mutation Y58N is the only mutation in the acceptor binding site. The loop mutations are located further away but were hypothesized to affect the water network in the active site [11]. In the present work, the mutations were split into three groups and systematic combination of these resulted in a total of seven mutants which were constructed, expressed, and tested experimentally. This led to identification of two novel enzymes with improved trans-sialidase activity and reduced hydrolase activity.

## Materials and methods

### Substrates

3’SL was purchased from Carbosynth (Compton, United Kingdom). The commercial casein glycomacropeptide (CGMP) product Lacprodan® CGMP-20 containing 5.7% (w/w) covalently linked sialic acid was a gift from Arla Foods amba (Viby, Denmark). Before use, low molecular weight impurities in the CGMP solution were removed by filtration using a 5 kDa membrane (Sartorius AG, Goettingen, Germany) as a technical precaution relating to High-performance anion exchange chromatography (HPAEC) analysis. All other chemicals were purchased from Sigma-Aldrich (Steinheim, Germany).

### DNA manipulations and strain construction

Construction of His_6_-tagged genes encoding TrSA_6mut_ and TrSA_13mut_ for secreted expression in *P*. *pastoris* was described previously [12,11]. These genes were used as templates for introduction of additional mutations using the QuikChange II Site-Directed Mutagenesis Kit (Agilent, CA, USA) and mutagenic primers (Table 1). All plasmids were sequenced to confirm the mutations and the integrity of the genes. Plasmids encoding the different enzyme variants were propagated in *Escherichia coli* DH5α, cultured at 37°C while shaking in low salt LB medium (10 g/L tryptone, 5 g/L yeast extract and 5 g/L NaCl), supplemented with 25 μg/mL zeocin. *P*. *pastoris* X-33 was transformed with the *Mss*I-linearized vectors by electroporation following the manufacturer’s instructions (Invitrogen, CA, USA).

**Table 1:** List of primers used for mutagenesis. Mutagenic codons are indicated in bold.

Name	Sequence
T39A(I37L)_fwd	GGTTCATTCATTTAGA**TTA**CCA**GCT**ATCGTTAACGTAGATGGAGT
T39A(I37L)_rev	ACTCCATCTACGTTAACGAT**AGC**TGG**TAA**TCTAAATGAATGAACC
F59N_fwd	CTGATGCCAGATATGAGACATCA**AAC**GACAACTCCTTTATCGAA
F59N_rev	TTCGATAAAGGAGTTGTC**GTT**TGATGTCTCATATCTGGCATCAG
D285G_fwd	CAACTTCCAATCAACCC**GGT**TGTCAGAGTTCATTCGT
D285G_rev	ACGAATGAACTCTGACA**ACC**GGGTTGATTGGAAGTTG
G342A_fwd	ATTGGTGATGAAAACAGT**GCT**TACTCTTCCGTCCTATAC
G342A_rev	GTATAGGACGGAAGAGTA**AGC**ACTGTTTTCATCACCAAT
L37I_fwd	CGTGTGGTTCATTCATTTAGA**ATA**CCAACTATCGTTAACGTAG
L37I_rev	CTACGTTAACGATAGTTGG**TAT**TCTAAATGAATGAACCACACG
T39A(I37)_fwd	CGTGTGGTTCATTCATTTAGAATACCA**GCT**ATCGTTAACGTAGATGG
T39A(I37)_rev	CCATCTACGTTAACGAT**AGC**TGGTATTCTAAATGAATGAACCACACG

### Enzyme production and purification

Protein synthesis was performed by cultivation of *P*. *pastoris* X-33 harboring pPICZαC with the mutated genes in shake flasks for three days in accordance with the EasySelect™ Pichia Expression Kit (Invitrogen) protocol. Briefly, induction was carried out in 200 or 1000 mL BMMY (0.5% methanol) while shaking at 28°C. Protein synthesis was induced every 24 hours by addition of methanol to a final concentration of 0.5%. His_6_-tagged enzymes were purified as described previously [16]. Protein concentration was estimated by measurement of absorbance at 280 nm using extinction coefficients calculated using ProtParam [17].

### Trans-sialidase activity analysis

The donor substrate CGMP was purified as described previously [18] with the modification that all reaction mixtures were prepared in 20 mM phosphate-citrate buffer (pH 6.4). D-Lactose was added to the CGMP solution, to final D-lactose and CGMP concentrations of 7.5 g/L and 50.6 g/L, respectively. Enzyme preparations were pre-diluted to 57 or 5.7 μg/mL total protein with 20 mM phosphate-citrate buffer (pH 6.4). Reactions were started by addition of 185 μL pre-diluted enzyme solution to 515 μL substrate mix (both preheated at 30°C) and the resulting reaction mixtures, either 1.5 μg/mL (TrSA_5mut_, TrSA_8mut_, TrSA_12mut_, and TrSA_15mut_) or 15 μg/mL (TrSA_6mut_, TrSA_9mut_, TrSA_13mut_, and TrSA_16mut_) enzyme, 16 mM D-lactose, 37 g/L CGMP (corresponding to approximately 4 mM 3’-bound and 4 mM 6’-bound SA [19]), were incubated at 30°C with shaking (700 rpm). Note that Trypanosomal trans-sialidases are generally considered specific for α2,3-bound sialic acid and we have previously shown that TrSA_13mut_ does not act on 6’-sialyllactose [11] thus making it unlikely that 6’-bound SA would constitute a substrate. Reactions were terminated by heat inactivation at 90°C for 10 min and filtered through a 5 kDa Vivaspin filter (Sartorius) by centrifugation for 10 min at 5000 g and 4°C. Filtrates were analyzed by HPAEC-PAD as described below. As a control, reactions were performed using the same enzyme preparations after heat-inactivation at 90°C for 10 min. All reactions were done in duplicates. To estimate initial rates, the data points from 5 to 40 min in each replicate were fitted with a second degree polynomial function and the slopes at time 0 min were calculated.

### High-Performance Anion Exchange Chromatography (HPAEC-PAD)

Separation and quantification of reaction products (3’SL and SA) from biocatalytic reactions were carried out using a Dionex BioLC system consisting of a GS50 gradient pump, an ED50 electrochemical detector and an AS50 chromatography compartment coupled to an AS50 autosampler (Dionex Corp., Sunnyvale, CA) as described previously [18].

### Enzyme 3D models

Enzyme 3D models of TrSA mutants were generated using the software PyMol v1.3 (Schrödinger) by introduction of point mutations in a TrSA crystal structure (PDB 1N1T) [20]. A TcTS crystal structure (PDB 1S0I) [7] was used as alignment reference.

## Results and discussion

### Design of TrSA mutants

**Fig 1:**
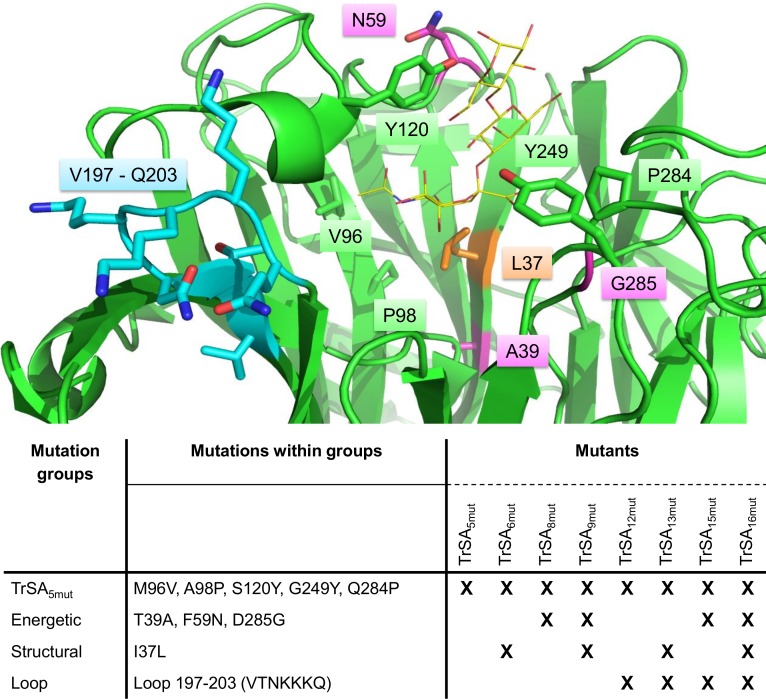
Mutations introduced in TrSA. The top panel displays a close-up of the active site with 3’SL docked (yellow, sticks). The 16 mutations introduced in TrSA_16mut_ are colored according to the various mutation groups: structural mutation (orange), energetic mutations (magenta), loop mutations (cyan) and mutations part of TrSA_5mut_ (green). The bottom panel offers an overview of the mutation groups and the specific mutations introduced in the different TrSA mutant enzymes.

By combining the findings of previous experimental [10,11] and computational studies [13] we aimed to design mutants of TrSA with improved trans-sialidase activity and/or reduced hydrolytic activity. As a starting point, the mutant TrSA_5mut_ was chosen as it was previously shown to contain the minimal amount of residues necessary for conferring detectable trans-sialidase activity to the enzyme [10]. Next, we grouped the mutations based on expected functionality, thereby obtaining three groups of mutations that were combined in the TrSA_5mut_ backbone. The first group was termed “energetic mutations”, and consisted of T39A, F59N, and D285G, that based on energy decomposition analysis were predicted to lower the stability of the CI thus promoting trans-sialidase activity [13]. The second group was termed “structural mutations” and consisted of I37L and G342A that were predicted to improve the mobility of the nucleophile [13]. However, due to the fact that eight enzyme variants with the G342A mutation were expressed at levels insufficient for enzyme activity measurements, the group was subsequently reduced to only include I37L. The third group was termed “loop mutation” and consisted of the highly positively charged seven amino acid motif (VTNKKKQ) that, when introduced in TrSA_6mut_, led to a reduced hydrolase activity without negatively affecting the trans-sialidase activity [11]. Introduction of the different combinations of mutation groups into TrSA_5mut_ led to the design of seven mutant enzymes containing 1 to 11 additional point mutations as summarized in Fig 1.

### Enzyme production in *P*. *pastoris*

To test the designed enzymes, we constructed genes encoding the enzyme variants and expressed them in the yeast *P*. *pastoris*. Expression levels of successfully expressed mutant variants were in the range of 100–850 μg/L, which was sufficient for the analyses performed in this study. As mentioned in the previous section, extremely low or no expression was observed for eight variants carrying the mutation G342A alone or in combination with I37L, respectively. This was also observed previously when expressed in *E*. *coli* [10]. In contrast, we observed that variants with only the I37L mutation expressed well in *P*. *pastoris* [12]. In order to find an explanation for the unsuccessful expression of variants carrying the G342A mutation, we compared the crystal structures of TrSA [20] and TcTS [7], and observed a significant structural difference between the two enzymes that could potentially explain the observed low production (S1 Fig).

### Trans-sialidase activity assessment

**Fig 2:**
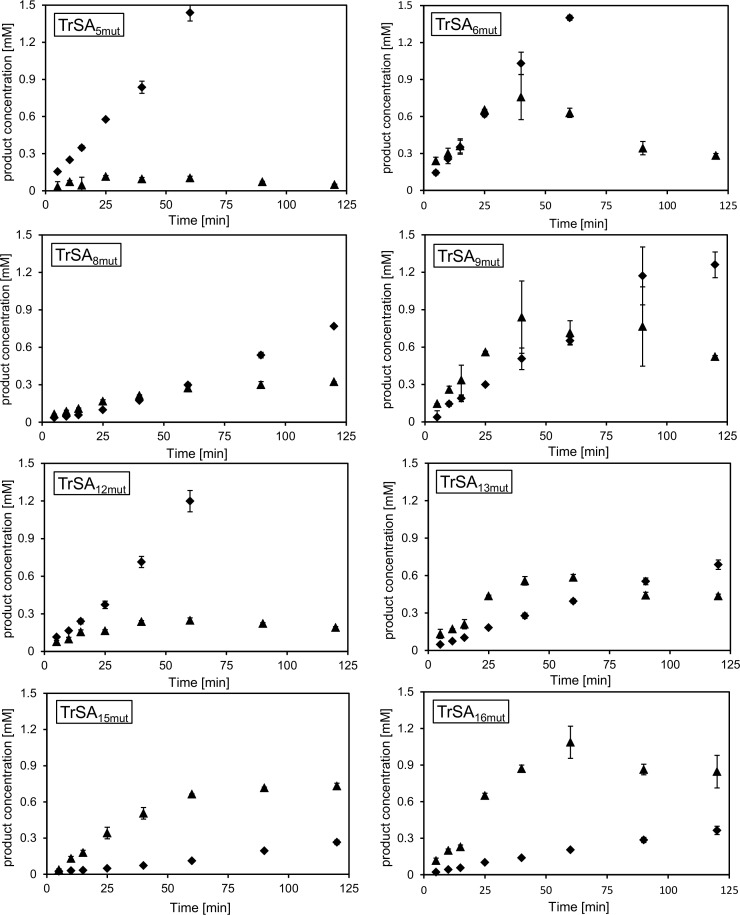
Time course experiments for 3’SL production. Time course experiments using the different TrSA mutants: TrSA_5mut_, TrSA_6mut_, TrSA_8mut_, TrSA_9mut_, TrSA_12mut_, TrSA_13mut_, TrSA_15mut_, and TrSA_16mut_. The concentration of SA (diamonds) and 3’SL (triangles) at the respective sampling times is shown. Mutants with the structural mutation (TrSA_6mut_, TrSA_9mut_, TrSA_13mut_, and TrSA_16mut_) were assayed using 15 μg/mL enzyme and those without (TrSA_5mut_, TrSA_8mut_, TrSA_12mut_, and TrSA_15mut_) were assayed using 1.5 μg/mL. TrSA_5mut_, TrSA_6mut_ and TrSA_12mut_ had a very fast initial hydrolysis (SA), whereas reasonable amounts of 3’SL were generated by the remaining enzymes. TrSA_15mut_ and TrSA_16mut_, however exhibited the highest ratio of 3’SL:SA.

To evaluate the novel TrSA mutant enzymes, we used the commercially interesting sialyl-donor substrate CGMP in combination with D-lactose as acceptor. As expected for bimolecular reactions, we previously demonstrated that the use of higher acceptor concentrations improves trans-sialidase activity, even for enzymes with relatively high hydrolase activity [12]. In order to highlight differences in trans-sialidase activity between the enzyme variants assessed in this paper, D-lactose was used at a lower concentration (with a donor:acceptor ratio of 1:4) in time course experiments (Fig 2). We initially optimized the enzyme concentration based on the activity of TrSA_6mut_. However, for the four variants without the structural mutation I37L (TrSA_5mut_, TrSA_8mut_ TrSA_12mut_ and TrSA_15mut_; Fig 1) the activity was too high to observe the initial 3’SL formation rate. Therefore reactions for variants with I37 were done with a 10-fold lower enzyme concentration compared with those variants carrying the I37L mutation. Overall, it was observed that all of the mutants produced more 3’SL than the parent enzyme TrSA_5mut_. For most variants, only slight improvements in 3’SL formation were observed, whereas TrSA_15mut_ and TrSA_16mut_ showed dramatic increases in trans-sialidase activity (Fig 2).

The Trypanosomal trans-sialidases and mutants of the *T*. *rangeli* sialidase have previously been reported to be specific for α2,3-sialylation of the terminal sugar [11,12]. From the HPAEC-PAD analysis it was also evident that 3’SL was the dominant sialylation product for all enzyme variants (S2 Fig). A minor sialylation product, tentatively 3-sialyllactose (3SL) [21], was seen for all enzyme variants. TrSA_16mut_ reactions contained only trace amounts of this compound.

Improved yield of trans-sialylation product can be a consequence of improved trans-sialidase activity and/or reduced hydrolase activity. To investigate this, we estimated the initial rates of sialic acid release and 3’SL synthesis from the time course experiments (Table 2). From the specific initial rates, it is evident that the enzyme variants with the structural mutation I37L, all exhibit a lower overall activity, while it only in certain contexts appear to substantially improve the trans-sialidase/hydrolase ratio. When considering the three groups of mutations (structural, energetic, and loop mutations) individually, it was observed that the loop mutation led to a 2.3-fold higher 3’SL:SA ratio compared to TrSA_5mut_, whereas the structural and energetic mutations improved the ratio 4.4- and 6.8-fold, respectively. As was evident from the time course experiment, the most proficient enzymes where obtained by combining the loop and energetic mutations (with and without the structural mutation), TrSA_15mut_, and TrSA_16mut_, which led to a 13-fold improvement of the 3’SL:SA ratio.

**Table 2:** Estimated specific initial rates for 3’SL formation and SA release.

Enzyme	Trans-sialidase activity	Hydrolase activity	3’SL:SA ratio	Fold change^1^
	[pmol 3’SL/(min*μg protein)]	[pmol SA/(min*μg protein)]		
TrSA_5mut_	5.87±0.24	17.74±0.15	0.33	1
TrSA_6mut_	2.29±0.03	1.58±0.00	1.45	4.4
TrSA_8mut_	6.18±0.46	2.77±0.17	2.23	6.8
TrSA_9mut_	1.69±0.14	0.81±0.08	2.09	6.3
TrSA_12mut_	7.47±0.93	9.89±0.69	0.76	2.3
TrSA_13mut_	1.28±0.02	0.51±0.01	2.53	7.7
TrSA_15mut_	8.56±1.41	1.87±0.07	4.57	13.8
TrSA_16mut_	1.41±0.09	0.31±0.01	4.48	13.6

^1^ Fold-change of the 3’SL:SA ratio compared to TrSA_5mut_

### Evaluation of time course experiments

TrSA_5mut_, TrSA_6mut_ and TrSA_13mut_ have all been characterized previously, but this is the first study in which these mutants have been compared using the same experimental conditions and kinetic modelling. TrSA_6mut_ was first described as a mutant with a 12-fold improved trans-sialidase activity compared to the TrSA_5mut_ [10]. The enzymes were assayed at one time point analyzing product concentrations in the μM range using 3’SL as donor and C^14^-labeled D-lactose as acceptor [10]. In trans-glycosylation reactions characterized by an initial product build-up followed by product degradation, we find that the time course experiment is more robust to changes in enzyme concentration and sampling time. Using our setup with CGMP as donor substrate, we observed that the product to hydrolysis ratio was greatly enhanced for TrSA_6mut_ and this led to an about 7-fold higher maximal product concentration (Fig 2) which largely support the conclusion of Paris and co-workers [10]. In our previous work, TrSA_6mut_ and TrSA_13mut_ were compared based on a fluorometric time course assay using an artificial acceptor substrate [11]. In the study presented here, using a more relevant acceptor substrate (D-lactose), we confirmed that TrSA_13mut_ performs better than TrSA_6mut_ which was mediated via both a reduction in trans-sialidase activity but also a substantially higher reduction in hydrolytic activity of TrSA_13mut_ when compared to the TrSA_6mut_.

The study of Pierdominici-Sotille and co-workers suggested five mutations to be introduced in TrSA_5mut_ that should yield an enzyme with high trans-sialidase activity comparable to that of the TcTS [13]. This was not confirmed experimentally in their publication, and in our hands, production of TrSA_10mut_ was not possible due to the destabilizing mutation G342A. The closest related, expressed enzyme TrSA_9mut_ (lacking G342A) showed slightly higher 3’SL production compared to TrSA_6mut_ and this appeared to be mainly due to reduced hydrolysis activity in TrSA_9mut_. This could indicate, that although the bioinformatics approach was able to identify amino acid mutations relevant to the trans-sialidase reaction, it overestimated the impact of these mutations.

With respect to 3’SL production, the variant TrSA_16mut_ containing all three groups of mutations and TrSA_15mut_ without the I37L mutation displayed a substantially higher maximal 3’-SL production than TrSA_13mut_ and variants with fewer mutations. Both enzymes exhibited a similar trans-sialidase to hydrolase ratio but whereas TrSA_15mut_ had a slightly increased trans-sialidase activity, it was reduced 4-fold in TrSA_16mut_.

### Molecular interpretation of mutations

#### The structural mutations

The structural mutation I37L greatly reduced the activity for both the production of 3’SL and free sialic acid, irrespective of the background. It appears unlikely that the lower activity is solely due to an increased flexibility of the nucleophile. The reduced activity of the mutants could arise from a rearranged nucleophile, which results in a covalent intermediate less susceptible to an attack from both water and acceptor, e.g. (i) due to steric shielding (although Ile and Leu are isomers) or (ii) due to a less optimal interaction of substrates and the catalytic residues due to a conformation change in the presence of Leu compared to Ile.

#### The energetic mutations

The energetic mutations when introduced in TrSA_5mut_ did not affect the trans-sialidase activity but also reduced the hydrolysis activity by a factor of 6.4, thus making TrSA_8mut_ better at producing 3’SL than TrSA_5mut_. When introduced into other TrSA-variants, the energetic mutations generally had limited effect on trans-sialidase activity, led to more modest decreases in the hydrolytic activity. The higher trans-sialidase activity could be explained by the proposed destabilization of the covalent intermediate [13], whereas the decreased product hydrolysis could be (i) due to changes in the water accessibility or (ii) an alteration of the conformation in the substrate/product bound state, which disfavours hydrolysis by e.g. a better indirect exclusion of water from the active site.

#### The loop mutation

Introduction of the loop mutations generally had little effect on the trans-sialidase activity, but rather led to a reduction in hydrolysis when compared with the respective variants without the loop. The loop mutation has been suggested previously to influence the water network surrounding the active site, thereby hindering water from attacking the covalent intermediate [11]. This is in good agreement with the lower hydrolase activity observed when combining the loop mutations with either structural or energetic mutations. When combined with both of the mutation groups, it appeared however to have a negative effect on overall activity reducing hydrolase activity more dramatically, but also decreasing the trans-sialidase activity.

## Conclusions

This study combined the results of three previous mutagenesis and bioinformatics studies on TrSA with the aim of designing enzymes with improved trans-sialidase activity and/or reduced hydrolytic activity. Three groups of mutations, based on suggested mechanistic properties, were combined systematically to design seven TrSA mutant variants. The seven variants were tested in time course experiments to evaluate the effects of the individual mutation groups. All seven expressed mutants exhibited enhanced trans-sialidase to hydrolase activity ratios compared to the parent enzyme TrSA_5mut_. Based on the time course experiments, two enzymes, TrSA_15mut_ and TrSA_16mut_, stood out as drastically improved with respect to 3’SL production. These two enzymes exhibited different kinetics due to the I37L mutation in TrSA_16mut_, which led to reduced overall activity compared to TrSA_15mut_. In conclusion, the two enzyme variants, TrSA_15mut_ and TrSA_16mut_, constitute further improvements in the development of trans-sialidases based on the TrSA_5mut_, TrSA_6mut_ and TrSA_13mut_ sialidase and should prove useful for the preparation of sialylated biomolecules.

## Supporting information

S1 Fig Inspection of 3D structures of TrSA and TcTS around TrSA G342. Mutation of G342 in TrSA led to low/no expression. The figure shows the conformation of the carboxylic oxygens of TcTS S340 and TrSA S341 adjacent to G341/342 in each of the two enzymes TcTS and TrSA. (Left) In TcTS the residue assumes a conformation with a disallowed phi/psi angle set and we suggest that the mutation of the adjacent residue (G341A) might lead to a similar phi/psi angle set of the S340 in the TrSA since no other differences can be observed in the structures around S340/S341 (Right). The fact that the disallowed conformation of S340 in TcTS can exist might be due to an array of stabilizing mutations introduced by evolution. Such higher stability could explain why TcTS is able to cope with the destabilization caused by S340. In contrast, our mutagenesis approach would not introduce such compensatory mutations that would enable the presence of the disallowed conformation.
(TIF)

S2 Fig HPAEC-PAD chromatograms of trans-sialylation products. Chromatograms from the analysis of samples at the time of transient maximal 3’SL yield for the enzyme variants TrSA_5mut_ (25 min), TrSA_6mut_ (40 min), TrSA_8mut_ (120 min), TrSA_9mut_ (40 min), TrSA_12mut_ (60 min), TrSA_13mut_ (60 min), TrSA_15mut_ (120 min), and TrSA_16mut_ (60 min). The hydrolysis product SA and the trans-sialidase products 3’SL and the minor sialylation compound (*) are marked.
(TIF)
