# Supplementary figures and images for: Design of Trypanosoma rangeli sialidase mutants with improved trans-sialidase activity
Source: PLoS One. 2017 Feb 3;12(2):e0171585. doi: 10.1371/journal.pone.0171585 (PMC5291517; doi:10.1371/journal.pone.0171585)

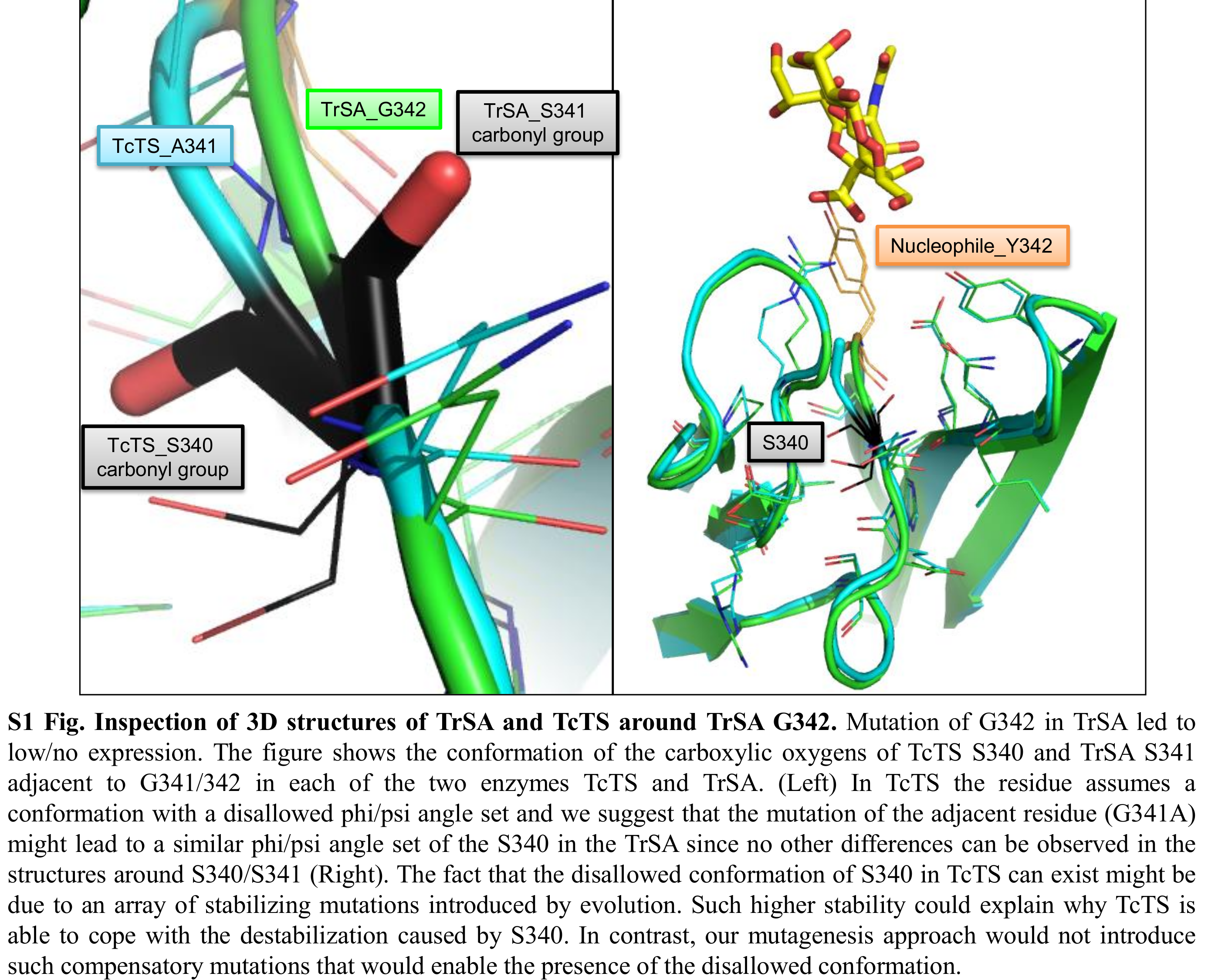

Supplement: S1 Fig — Mutation of G342 in TrSA led to low/no expression. The figure shows the conformation of the carboxylic oxygens of TcTS S340 and TrSA S341 adjacent to G341/342 in each of the two enzymes TcTS and TrSA. (Left) In TcTS the residue assumes a conformation with a disallowed phi/psi angle set and we suggest that the mutation of the adjacent residue (G341A) might lead to a similar phi/psi angle set of the S340 in the TrSA since no other differences can be observed in the structures around S340/S341 (Right). The fact that the disallowed conformation of S340 in TcTS can exist might be due to an array of stabilizing mutations introduced by evolution. Such higher stability could explain why TcTS is able to cope with the destabilization caused by S340. In contrast, our mutagenesis approach would not introduce such compensatory mutations that would enable the presence of the disallowed conformation. (TIF) [file pone.0171585.s001.tif]

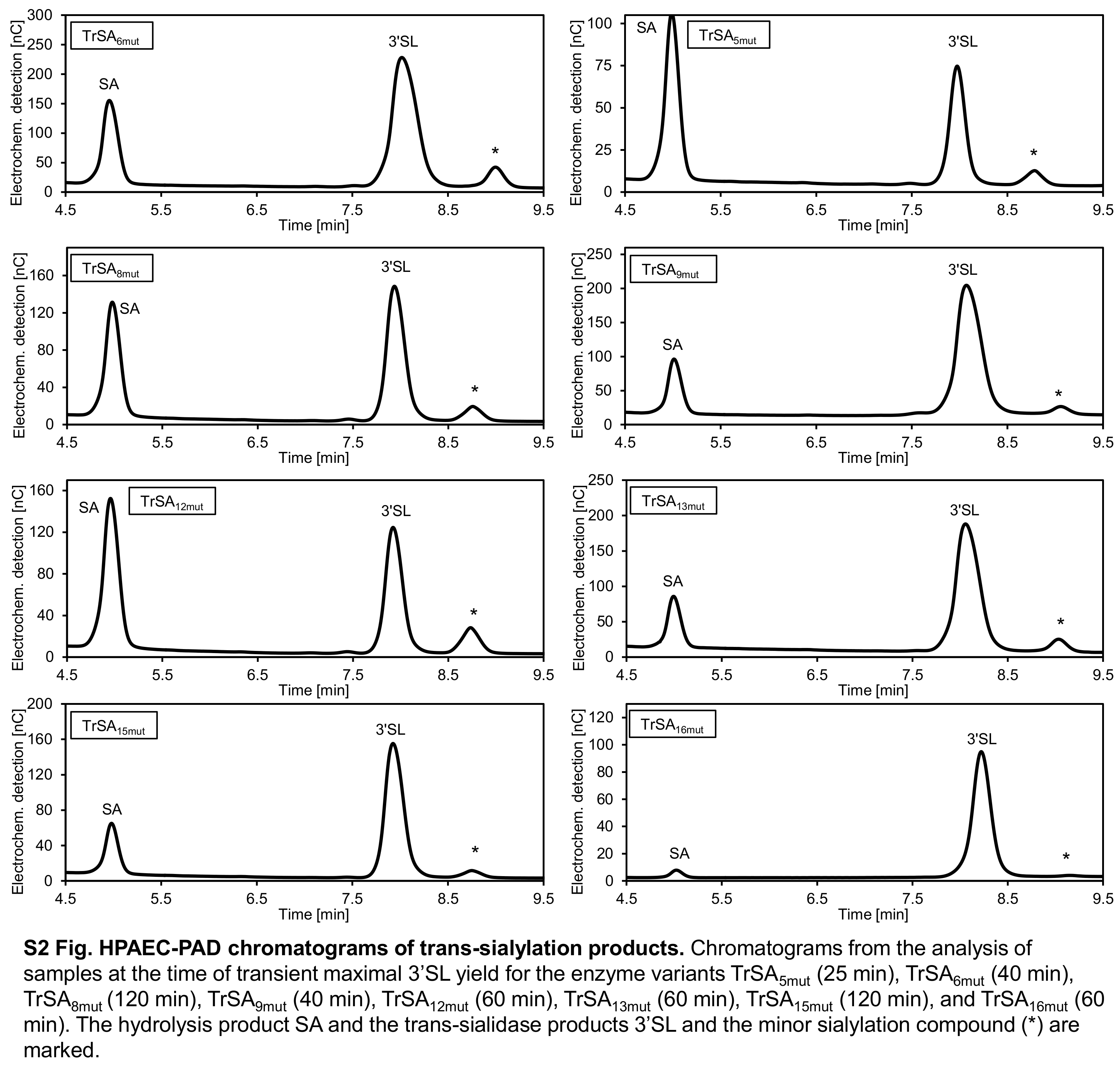

Supplement: S2 Fig — Chromatograms from the analysis of samples at the time of transient maximal 3’SL yield for the enzyme variants TrSA5mut (25 min), TrSA6mut (40 min), TrSA8mut (120 min), TrSA9mut (40 min), TrSA12mut (60 min), TrSA13mut (60 min), TrSA15mut (120 min), and TrSA16mut (60 min). The hydrolysis product SA and the trans-sialidase products 3’SL and the minor sialylation compound (*) are marked. (TIF) [file pone.0171585.s002.tif]
